# Economic Deterioration and Social Factors Affecting Mental Health During the COVID-19 Pandemic in Japan: Web-Based Cross-Sectional Survey

**DOI:** 10.2196/65204

**Published:** 2025-06-10

**Authors:** Kentaro Hori, Yosuke Yamada, Hideyuki Namba, Misaka Kimura, Hiroyuki Fujita, Heiwa Date

**Affiliations:** 1 Faculty of Data Science, Shiga University, 1-1-1 Banba, Hikone City, 522-8522, Japan, 81 09080737007; 2 Department of Physical Activity Research, National Institutes of Biomedical Innovation, Health and Nutrition, Settsu City, Japan; 3 Sports and Health Education Division, Center for Education in Liveral Arts and Sciences, Osaka University, Toyonakashi City, Japan; 4 Institute for Active Health, Kyoto University of Advanced Science, Kyoto City, Japan; 5 Faculty of Bioenvironmental Science, Kyoto University of Advanced Science, Kyoto City, Japan

**Keywords:** COVID-19, mental health, economic deterioration, social support, Japan

## Abstract

**Background:**

The socioeconomic impact of the COVID-19 pandemic has severely affected individuals’ mental health. However, the factors that mitigate or exacerbate the mental health effects of economic deterioration remain underexplored.

**Objective:**

This paper analyzes survey data from the second wave of the COVID-19 pandemic in Japan, a period during which women workers were reported to be economically and psychologically vulnerable. The analysis examined factors that mitigate or amplify the impact of COVID-19-induced economic deterioration on mental health, testing 3 hypotheses based on the conservation of resources theory and the stress buffering model: the negative impact of economic deterioration on mental health is greater for individuals with less social support compared to those with more social support (hypothesis 1); the negative impact of economic deterioration on mental health is greater for individuals experiencing more negative interactions compared to those experiencing fewer (hypothesis 2); and the buffering effect of social support is stronger in women than in men, with women receiving less social support experiencing greater mental health impacts from economic deterioration (hypothesis 3).

**Methods:**

A web-based survey was conducted by an internet research company in Japan from June to July 2020. A balanced sample of 250 men and 250 women was recruited from each of the following age groups: 20-29, 30-39, 40-49, 50-59, 60-69, and 70-79 years. The analysis focused on working men and women aged 20‐50 years (n=1238). Psychological distress was measured using the K6 scale. Economic deterioration was defined as a decrease in income compared to the prepandemic levels, and scales for social support and negative interactions were included. Logistic regression analysis was performed, using K6≥9 as the dependent variable, with interaction terms for each hypothesis sequentially incorporated.

**Results:**

In the best-fitting model determined by the Bayesian Information Criterion, a significant association was observed between the interaction of COVID-19-induced economic deterioration and social support with K6 scores (odds ratio [OR] 0.90, 95% CI 0.81‐0.99). However, in other models, the interaction between economic deterioration and negative interactions (OR 1.01, 95% CI 0.90‐1.13) as well as the 3-way interaction involving economic deterioration, social support, and gender (OR 1.13, 95%CI 0.92‐1.39) were not significant. The average marginal effect of economic deterioration was statistically significant for social support scores ranging from 4 to 10. The average marginal effect was 0.11 when social support was 4 (95% CI 0.03‐1.20; *P*=.009) and 0.028 when social support was 10 (95% CI 0.00‐0.06; *P*=.047).

**Conclusions:**

The adverse impact of economic deterioration on mental health was more pronounced among individuals with lower levels of social support. These findings support hypothesis 1.

## Introduction

The spread of COVID-19 forced people to change their lifestyles, raising significant concerns about mental health deterioration. Those who faced unemployment or had a history of chronic or mental illnesses were particularly susceptible to worsening mental health [1-5]. The socioeconomic downturn caused by the pandemic contributed to an increase in suicides, with unemployment, economic insecurity, and poverty having profound effects [2,3,5]. In Japan, where the suicide rate was already high before the pandemic, the number of suicides had been decreasing but began to rise again after the outbreak [6]. Analyses of suicide and depression risk factors in Japan identified economic deterioration from socioeconomic stagnation as a key contributor [7,8].

During the pandemic, individuals were exposed to negative events such as economic decline, while restrictions on interpersonal relationships reduced available social support, adversely affecting mental health [1,9-12]. For example, mothers raising children faced increased stress due to limited contact with grandparents, a crucial source of support [2]. Conversely, individuals with stronger social support were better able to manage loneliness under these restrictions [13,14], alleviate anxiety about the pandemic among pregnant women [15], and mitigate the impact of work-related stress on mental health [16].

Negative interactions, such as domestic violence during stay-at-home orders [17] and deteriorating relationships with family or partners [18], also emerged, highlighting new challenges in close relationships. Previous studies have revealed that such interactions negatively impacted mental health [19,20].

As outlined, previous studies have demonstrated that social support and negative interactions directly influenced mental health during the pandemic. However, the role of factors mitigating or exacerbating the negative impact of economic deterioration on mental health remains insufficiently explored, particularly in the context of limited interpersonal interactions. Social support and negative interactions, as discussed below, may serve as such factors.

Incorporating the conservation of resources hypothesis and the stress buffering model may provide new insights into social support during periods of widespread infection. Hobfoll’s conservation of resources hypothesis defines resources as elements valued by individuals in society, including material resources (eg, money and housing), social resources (eg, social support and status), and psychological resources (eg, personal achievement and autonomy). The hypothesis posits that people feel stress when they lose or face the potential loss of valued resources, as these are essential for daily life and goal achievement [21-23]. For example, loss of income or employment (material resources) threatens stability and induces anxiety and stress. A study in Israel during the pandemic using the conservation of resources hypothesis found that individuals who experienced economic deterioration and loss of resources such as income and socioeconomic status showed declines in mental health [24].

In addition, the conservation of resources theory suggests that individuals facing resource loss may use other resources, such as social support, to cope and reduce stress. Social support, in particular, is a vital resource individuals rely on in emergencies to prevent further resource loss [21]. Those with stronger social support are better equipped to manage stress through assistance and information from others [22].

The stress buffering model provides a practical and statistical explanation for the benefits of social support. According to this model, social support not only has a direct positive effect on mental health but also serves as an indirect protective factor, alleviating stress and psychological burden during stressful events [25-29]. The model posits that social support helps resolve stressful situations and reduces the perception of stress through positive interpersonal relationships. Studies testing this model typically use interaction terms between stressful events and social support as explanatory variables [25,28,29].

Based on these theories, it can be anticipated that social support may mitigate the negative impact of economic deterioration on mental health during the COVID-19 pandemic. To verify this, it is necessary to analyze the interaction term between economic deterioration and social support. However, previous studies have focused primarily on the main effects of economic deterioration and social support, without considering their interaction [7,17,30].

Conversely, negative interactions tend to have the opposite effect of social support, with experiences such as criticism or excessive demands from others worsening mental health [26,31]. According to the conservation of resources theory, economic deterioration, which signifies the depletion of critical resources such as income, induces stress. Under such circumstances, negative interactions are likely to amplify the risk of mental health deterioration. However, studies on negative interactions during the pandemic have not analyzed the interaction between economic deterioration and negative interactions, leaving their synergistic effects unexamined [19,20].

This paper addresses these gaps by analyzing these interaction terms and testing the following hypotheses.

First, hypothesis 1: the negative impact of economic deterioration due to the COVID-19 pandemic on mental health is stronger for individuals with less social support compared to those with more social support.

Second, hypothesis 2: the negative impact of economic deterioration due to the COVID-19 pandemic on mental health is stronger for individuals with more negative interactions compared to those with fewer negative interactions.

Furthermore, this study examined hypotheses related to the high suicide rate among women during the pandemic in Japan. The first wave of the pandemic occurred in April 2020, followed by a larger second wave beginning in July 2020. During the second wave, women’s mental health deteriorated significantly, with the female suicide rate increasing approximately 5 times that of men, marking a distinct shift [8,32,33]. This deterioration was partly attributed to the substantial economic damage in industries predominantly employing women, such as service, retail, and tourism [8,33]. Historically, the suicide rate among Japanese women has not exceeded that of men, but during this period, women in the workforce may have been more likely than men to face economic and psychological vulnerability.

Drawing on the conservation of resources theory, female workers likely perceived income loss as a significant threat during the second wave. Among these women, those with strong social support may have been better equipped to cope with the stress of income loss, benefiting from its buffering effects. Therefore, this study used data from a survey conducted in Japan between June and July 2020, which covers the second wave. It analyzed a 3-way interaction by adding a gender variable to the interaction term of hypothesis 1 to test the third hypothesis: the buffering effect of social support is more pronounced in women than in men. Specifically, among women, the negative impact of economic deterioration due to the COVID-19 pandemic on mental health is stronger for those with less social support than for those with more social support.

This paper examined the 3 hypotheses above, focusing on the factors that mitigate or amplify the negative impact of economic deterioration caused by COVID-19 on mental health, and to clarify how social support and negative interactions function as such factors. This understanding is critical for developing targeted interventions and policies to support mental health during future crises.

## Methods

### Data Source

This study used primary data from a web-based survey conducted among residents of Japan. The survey was administered in a questionnaire format through Cross Marketing Inc, a Japanese internet research company. Cross Marketing Inc and its partner companies maintain an active panel of over 5 million people, who have registered their sociodemographic information in the company’s database and responded to at least one survey within the past year [34].

Data collection was conducted from June to July 2020 using Cross Marketing Inc’s web-based response system. The company provided respondents with a survey URL via email requesting their participation. First, individuals who agreed to participate underwent a screening survey regarding their demographic attributes, such as gender and age (n=19,301). Next, stratified sampling was performed, evenly allocating 250 men and 250 women from each age group (20s, 30s, 40s, 50s, 60s, and 70s), resulting in a total sample size of 3000 participants. This nonprobabilistic sampling was used to minimize data shortages for specific age and gender groups; however, it has limitations in ensuring representativeness. Respondents were selected in order of earliest response, and the survey continued until the target number of participants was reached, excluding inconsistent or invalid responses. The sample size was determined primarily based on feasibility and financial constraints.

In this study, economic deterioration was considered a key explanatory variable. In Japan, many individuals in their 20s to 50s work full-time and earn income, making them more susceptible to economic issues. Therefore, samples from individuals in their 60s and 70s were excluded, leaving a dataset of 2000 respondents.

Economic deterioration was analyzed based on changes in personal income, which required focusing on income earners. Respondents who had been unemployed before the pandemic and remained unemployed at the time of the survey (including full-time homemakers) were excluded. Those who were employed before the pandemic but unemployed during the survey period were also excluded due to their small number (n=17). These exclusions reduced the sample to 1513 respondents.

Following listwise deletion for missing values, the final dataset included 1238 respondents, with most exclusions resulting from non-responses to the household income question (n=254). In total, 18.2% (275/1513; 275 excluded) of the sample was excluded in this process.

### Measurements

The K6 scale was used to assess mental health, serving as the dependent variable. This scale measures psychological distress over the past 30 days with 6 items rated on a 5-point scale, producing total scores from 0 to 24, with higher scores indicating greater distress [35]. Furukawa et al [36] validated the reliability of the K6 scale in the Japanese population. Following their methodology, a cutoff score of ≥9 was used to screen for mood and anxiety disorders.

The primary independent variables for hypothesis testing were economic deterioration due to COVID-19, social support, and negative interactions. Economic deterioration was measured by asking, "How has your income (total pre-tax income from work and non-work sources) changed as of April compared to before the COVID-19 pandemic? Please select the option that best describes your situation. If your salary is yet to be confirmed (eg, not yet deposited), please answer based on your expectations.” The response options were: 1=increased (expected to increase), 2=slightly increased (expected to slightly increase), 3=almost unchanged, 4=slightly decreased (expected to slightly decrease), and 5=Decreased (expected to decrease). The response distribution was: 1 (n=21), 2 (n=37), 3 (n=735), 4 (n=197), and 5 (n=248). Categories 1 and 2 were combined with Category 3 and recorded as 0, while Categories 4 and 5 were combined and recorded as 1, resulting in binary variables. The correlation between economic deterioration and household income (a continuous variable) was weak (*r*=−0.15).

Social support was assessed using a 4-item, 4-point scale, with total scores ranging from 4 to 16, with higher scores indicating greater social support. The items were: “How much do your friends really care about you?” “How much do they understand your feelings?” “How much can you rely on them for help if you have a serious problem?” and “How much can you open up to them if you need to talk about your worries?” Response options were: 1=not at all, 2=a little, 3=some, and 4=a lot [37,38]. The Cronbach α was 0.90.

Negative interactions were measured using a 4-item, 4-point scale, with total scores ranging from 4 to 16, where higher scores indicated more negative interactions. The items were: “How often do your friends make too many demands on you?” “How often do they criticize you?” “How often do they let you down when you are counting on them?” and “How often do they get on your nerves?” Response options were: 1=never, 2=rarely, 3=sometimes, and 4=often [37,38]. The Cronbach α was 0.84.

The control variables used in this study were sex (1=female and 0=male), age, marital status (1=married, 0=single, divorced, or widowed), education level (with 3 categories: university or graduate school; junior college, technical college, or specialized training school; and high school or middle school), last year’s household income (log-transformed for analysis), employment conditions (regular employment, nonstandard employment, and self-employment), presence of chronic illness (1=no and 0=yes), and residential area (1=urban and 0=rural).

### Ethical Considerations

This study was approved by the ethics committees of the Kyoto University of Advanced Science (KUAS 20‐3) and the National Institutes of Biomedical Innovation, Health, and Nutrition (NIBIOHN 202).

Informed consent was obtained on the research company Cross Marketing Inc’s website before participants completed the survey. Participants were presented with an information sheet on the web-based survey landing page, and only those who confirmed that they had read the information sheet and agreed to participate were included in the study.

Before transferring the data to researchers, the research company removed participants’ names, addresses, contact information, and any other details that could potentially be used to identify individuals.

Subjects registered with the research company could receive point rewards for participating in the survey (Cross Marketing Inc does not publicly disclose the number of points respondents receive for completing surveys).

### Statistical Analyses

A logistic regression model was used to evaluate the relationship between economic deterioration, social support, negative interactions, and mental health. Control variables included sex, age, marital status, education level, last year’s household income, employment conditions, presence of chronic illness, and residential area. In addition, interaction terms between economic deterioration and social support, as well as between economic deterioration and negative interactions, were added sequentially. A final model with a 3-way interaction term among economic deterioration, social support, and the female dummy variable was also examined. Statistical analyses were conducted using R (version 4.2.2; R Foundation for Statistical Computing). Listwise deletion was applied to data with missing values, yielding a final sample size of 1238 individuals.

## Results

Table 1 presents the descriptive statistics, while Table 2 shows the results of the logistic regression analysis. In Model 1 of Table 2, the main effects of economic deterioration due to COVID-19, social support, and negative interactions were evaluated. The odds ratio for economic deterioration due to COVID-19 was 1.49 (95% CI 1.12‐1.98), for social support it was 0.96 (95% CI 0.91‐1.00), and for negative interactions it was 1.22 (95% CI 1.15‐1.29).

**Table 1.** Descriptive statistics.

Characteristics	Female (n=505, 40.8%)	Male (n=733, 59.2%)	Total (N=1238)
Age (years), n (%)			
20-39	131 (25.9)	154 (21)	285 (23)
30-39	125 (24.8)	192 (26.2)	317 (25.6)
40-49	129 (25.5)	193 (26.3)	322 (26)
50-59	120 (23.8)	194 (26.5)	314 (25.4)
Marital status, n (%)			
Married	273 (54.1)	384 (52.4)	657 (53.1)
Single, divorced, or widowed	232 (45.9)	349 (47.6)	581 (46.9)
Educational background, n (%)			
University or graduate school	221 (43.8)	470 (64.1)	691 (55.8)
Junior college, technical college, or specialized training school	156 (30.9)	109 (14.9)	265 (21.4)
High school or middle school	128 (25.3)	154 (21)	282 (22.8)
Household income (¥), n (%)^a^			
Less than 2 million	43 (8.5)	50 (6.8)	93 (7.5)
2 to 3.99 million	146 (28.9)	149 (20.3)	295 (23.8)
4 to 6.99 million	142 (28.1)	265 (36.2)	407 (32.9)
7 to 9.99 million	120 (23.8)	152 (20.7)	272 (22)
Over 10 million	54 (10.7)	117 (16)	171 (13.8)
Employment conditions, n (%)			
Regular employment	252 (49.9)	566 (77.2)	818 (66.1)
Nonstandard employment	231 (45.7)	109 (14.9)	340 (27.5)
Self-employment	22 (4.4)	58 (7.9)	80 (6.5)
Chronic disease, n (%)			
Yes	107 (21.2)	191 (26.1)	940 (75.9)
No	398 (78.8)	542 (73.9)	298 (24.1)
Residence area, n (%)			
Urban	306 (60.6)	443 (60.4)	749 (60.5)
Rural	199 (39.4)	290 (39.6)	489 (39.5)
Economic deterioration due to COVID-19, n (%)			
Worsened	198 (39.2)	247 (33.7)	445 (35.9)
Not worsened	307 (60.8)	486 (66.3)	793 (64.1)
Social support			
Mean (SD)	10.0 (3.0)	9.1 (2.7)	9.5 (2.9)
Median (range)	10 (4‐16)	8 (4‐16)	9 (4‐16)
Negative interaction			
Mean (SD)	8.7 (2.6)	8.4 (2.3)	8.5 (2.8)
Median (range)	8.0 (4‐16)	8.0 (4‐16)	9.0 (4‐16)
K6 scale, n (%)			
K6 ≥9	145 (28.7)	176 (24.0)	321 (25.9)
K6 <9	360 (71.3)	557 (76)	917 (74.1)

a The exchange rate for Japanese yen (¥) to US dollars (US $) during the data collection period (June to July 2020): US $1=¥110.

**Table 2.** Results of binomial logistic regression (K6 ≥9 as the dependent variable).^a^

	Model 1^b^	
	OR^c^ (95% CI)	*P* value
Female (vs male)	1.13 (0.84-1.53)	.42
Age 20-29 (vs 50-59) years	3.90 (2.45- 6.28)	<.001
Age 30-39 (vs 50-59) years	2.96 (1.91- 4.63)	<.001
Age 40-49 (vs 50-59) years	2.12 (1.39-3.24)	<.001
Married (vs single, divorced, or widowed)	1.29 (0.94-1.78)	.12
University or graduate school (vs high school or middle school)	0.79 (0.55-1.13)	.19
Junior college, technical college, or specialized training school (vs high school or middle school)	0.86 (0.57-1.29)	.47
Household income	0.65 (0.52-0.81)	<.001
Nonstandard employment (vs regular employment)	1.04 (0.74-1.47)	.82
Self-employment (vs regular employment)	0.92 (0.50-1.64)	.79
No chronic disease (vs yes)	0.50 (0.36-0.69)	<.001
Urban (vs rural)	0.81 (0.61-1.08)	.15
Economic deterioration due to COVID-19 (vs not worsened)	1.49 (1.12-1.98)	.006
Social support	0.96 (0.91-1.00)	.08
Negative interaction	1.22 (1.15-1.29)	<.001

a The Bayesian Information Criterion (BIC) was 1378.15. After listwise deletion of missing data, 1238 respondents were included.

b The variance inflation factors (VIFs) ranged from 1.077 to 2.386 in Model 1.

c OR: adjusted odds ratio.

Table 3 and Table 4 present the results of the models with interaction terms. Model 2 includes the interaction term between economic deterioration due to COVID-19 and social support, Model 3 includes the interaction term between economic deterioration due to COVID-19 and negative interactions, Model 4 includes both interaction terms, and Model 5 includes the 3-way interaction term among economic deterioration due to COVID-19, social support, and the female dummy variable. The interaction between economic deterioration due to COVID-19 and social support was significant in Model 2 (OR 0.90, 95% CI 0.81‐0.99) and Model 4 (OR 0.89, 95% CI 0.81‐0.99). In Model 5, the 3-way interaction term was not significant. However, the interaction between economic deterioration due to COVID-19 and social support remained significant (OR 0.84, 95% CI 0.73‐0.96), confirming its effect. A supplementary analysis, not shown in Tables3  4, was conducted by adding the 3-way interaction term among economic deterioration due to COVID-19, negative interactions, and the female dummy variable to Model 5, which proved nonsignificant (OR 0.83, 95%CI 0.66‐1.05; *P*=.12).

**Table 3.** Results of binomial logistic regression including interaction terms (K6 ≥9 as the dependent variable) for Model 2 and Model 3.^a^

Variables	Model 2^b^			Model 3^b^		
	OR^c^ (95% CI)		*P* value	OR (95% CI)		*P* value
Female (vs male)	1.13 (0.83-1.52)		.44	1.13 (0.84-1.53)		.42
Age 20-29 (vs 50-59) years	3.95 (2.48-6.38)		<.001	3.90 (2.45-6.28)		<.001
Age 30-39 (vs 50-59) years	2.97 (1.92-4.66)		<.001	2.96 (1.91-4.63)		<.001
Age 40-49 (vs 50-59) years	2.11 (1.39-3.24)		.001	2.11 (1.39-3.24)		<.001
Married (vs single, divorced, or widowed)	1.29 (0.94-1.78)		.12	1.29 (0.94-1.78)		.12
University or graduate school (vs high school or middle school)	0.77 (0.54-1.11)		.17	0.79 (0.55-1.13)		.19
Junior college, technical college, or specialized training school (vs high school or middle school)	0.86 (0.57-1.29)		.46	0.86 (0.57-1.29)		.46
Household income	0.66 (0.52-0.82)		<.001	0.65 (0.52-0.81)		<.001
Nonstandard employment (vs regular employment)	1.04 (0.74-1.47)		.82	1.04 (0.74-1.47)		.82
Self-employment (vs regular employment)	0.95 (0.51-1.70)		.88	0.92 (0.50-1.64)		.79
No chronic disease (vs yes)	0.50 (0.36-0.68)		<.001	0.50 (0.36-0.69)		<.001
Urban (vs rural)	0.81 (0.60-1.08)		.14	0.81 (0.61-1.08)		.15
Economic deterioration due to COVID-19 (vs not worsened)	1.47 (1.11-1.96)		.008	0.96 (0.91-1.00)		.08
Social support	1.00 (0.94-1.06)		.94	1.48 (1.11-1.98)		.008
Negative interaction	1.22 (1.15-1.29)		.008	1.21 (1.13-1.30)		<.001
Economic deterioration due to COVID-19×social support	0.90 (0.81-0.99)		.03	—^d^		—
Economic deterioration due to COVID-19×negative interaction	—		—	1.01 (0.90-1.13)		.84

a The Bayesian Information Criterion (BIC) was 1380.62 for Model 2 and 1385.22 for Model 3. After listwise deletion of missing data, 1238 respondents were included for both models.

b The variance inflation factors (VIFs) ranged from 1.045 to 2.395 in Model 2, and 1.077 to 2.386 in Model 3.

c OR: adjusted odds ratio.

d Not applicable.

**Table 4.** Results of binomial logistic regression including interaction terms (K6 ≥9 as the dependent variable) for Model 4 and Model 5.^a^

Variables	Model 4^b^			Model 5^b^		
	OR^c^ (95% CI)		*P* value	OR (95% CI)		*P* value
Female (vs male)	1.12 (0.83-1.52)		.45	1.02 (0.70-1.49)		.90
Age 20-29 (vs 50-59) years	3.95 (2.48-6.39)		<.001	4.02 (2.51-6.52)		<.001
Age 30-39 (vs 50-59) years	2.97 (1.92-4.66)		<.001	3.07 (1.97-4.84)		<.001
Age 40-59 (vs 50-59) years	2.11 (1.39-3.24)		.001	2.11 (1.39-3.25)		<.001
Married (vs single, divorced, or widowed)	1.29 (0.94-1.78)		.12	1.28 (0.93-1.78)		.13
University or graduate school (vs high school or middle school)	0.77 (0.54-1.11)		.16	0.75 (0.53-1.09)		.13
Junior college, technical college, or specialized training school (vs high school or middle school)	0.86 (0.57-1.28)		.45	0.85 (0.57-1.28)		.45
Household income	0.66 (0.52-0.82)		<.001	0.66 (0.52-0.83)		<.001
Nonstandard employment (vs regular employment)	1.04 (0.73-1.47)		.83	1.04 (0.73-1.47)		.83
Self-employment (vs regular employment)	0.96 (0.51-1.71)		.88	0.94 (0.50-1.69)		.84
No chronic disease (vs yes)	0.50 (0.36-0.69)		<.001	0.50 (0.36-0.70)		<.001
Urban (vs rural)	0.81 (0.60-1.08)		.15	0.81 (0.60-1.08)		.15
Economic deterioration due to COVID-19 (vs not worsened)	1.46 (1.09-1.95)		.01	1.29 (0.88-1.90)		.19
Social support	1.00 (0.94-1.06)		.95	1.11 (1.01-1.21)		.03
Negative interaction	1.21 (1.12-1.30)		<.001	1.21 (1.13-1.31)		<.001
Economic deterioration due to COVID-19×social support	0.89 (0.81-0.99)		.03	0.84 (0.73-0.96)		.01
Economic deterioration due to COVID-19×negative interaction	1.03 (0.92-1.15)		.67	1.02 (0.91-1.14)		.79
Economic deterioration due to COVID-19×female	—^d^		—	1.27 (0.72-2.25)		.41
Social support×female	—		—	0.81 (0.71-0.92)		.001
Economic deterioration due to COVID-19×social support×female	—		—	1.13 (0.92-1.39)		.25

a The Bayesian Information Criterion (BIC) was 1387.55 for Model 4 and 1396.19 for Model 5. After listwise deletion of missing data, 1238 respondents were included for both models.

b The variance inflation factors (VIFs) ranged from 1.080 to 2.332 in Model 4, and 1.081 to 3.163 in Model 5.

c Odds ratio: adjusted odds ratio.

d Not applicable.

Finally, since the 2-way interaction term between economic deterioration due to COVID-19 and social support was significant, the average marginal effect of economic deterioration was calculated to elucidate this interaction. Model 2 demonstrated the best Bayesian Information Criterion, and thus, the average marginal effect was derived from this model.

**Figure 1.**
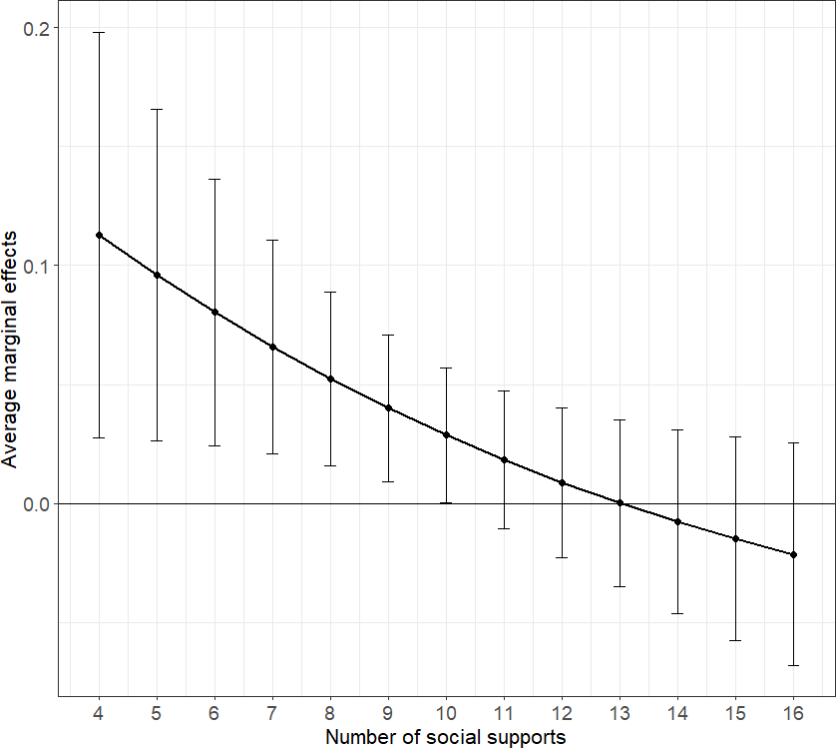
Average marginal effects of economic deterioration due to COVID-19 with 95% CI*.*

The average marginal effects of economic deterioration due to COVID-19 are shown in Figure 1. Based on the 95% CIs, the average marginal effects were significant when the number of social support items ranged from 4 to 10. The average marginal effect was 0.11 when social support was 4 (95% CI 0.03‐1.20*; P*=.009) and 0.028 when social support was 10 (95% CI 0.00‐0.06; *P*=.047). This finding suggests that the negative impact of economic deterioration on mental health is amplified when social support is low.

## Discussion

### Principal Findings

The primary finding of this study is that the negative impact of economic deterioration due to COVID-19 on mental health is amplified when social support is low, supporting hypothesis 1.

Previous studies have established that economic deterioration due to COVID-19 and social support affect mental health [2,7,14-17,30], but have examined the main effects of each variable. These analyses do not identify protective factors that mitigate the negative impact of economic deterioration. By contrast, this study suggests that social support functions as a protective factor. While social support has been widely recognized for its protective effects across various contexts [25-29], this study further confirms its role in mitigating the impact of economic deterioration during the pandemic.

The main effect of negative interactions was statistically significant in all models. Unlike previous studies [19,20], this study also analyzed the interaction between economic deterioration and negative interactions. However, this interaction term was not statistically significant, indicating that negative interactions do not exacerbate the negative impact of economic deterioration. Thus, hypothesis 2 was rejected. Nevertheless, the main effect of negative interactions remained consistently significant, aligning with previous studies [19,20] on the impact of negative interactions during the pandemic. Even before the pandemic, the influence of negative interactions was considered more significant than social support [31]. This suggests that the impact of negative interactions on mental health is consistently strong and should be recognized as a potent stressor, irrespective of the pandemic context.

In addition, building on previous studies that reported the economic and psychological vulnerability of female workers in Japan during the second wave of the COVID-19 pandemic [8,32,33], this study focused on women during this period. Specifically, drawing on the conservation of resources theory [21-23], hypothesis 3 was proposed to examine whether women with greater social support were better able to cope with this stressful event and benefit from its buffering effects. However, the 3-way interaction term required to test this hypothesis was not statistically significant, leading to its rejection.

By contrast, the persistent significance of the interaction between economic deterioration and social support suggests that the buffering effect of social support was effective regardless of gender. This result likely reflects the broader reality that, during this period, both men and women faced economic difficulties and social pressures, emphasizing the importance of social support for the mental health of both genders. In Japan, traditional gender roles, which position men as breadwinners and women as homemakers or caregivers, remain deeply ingrained [39]. These norms have long linked economic difficulties to suicide among working men in Japan, even before the outbreak [40,41]. During the pandemic, these issues continued to contribute to suicide rates [18], indicating that men faced significant pressures from both economic burdens and societal expectations tied to gender roles. Consequently, while women’s mental health deteriorated significantly during the second wave [8,32,33], men also faced the risk of losing critical resources, such as income. Therefore, social support likely served as an essential buffer for maintaining the mental health of both men and women.

### Limitations

This study has several limitations. First, its cross-sectional design restricted the ability to establish causal relationships. Individuals with preexisting poor mental health may struggle with work and interpersonal relationships, increasing their susceptibility to economic difficulties. As such, a bidirectional relationship, rather than a unidirectional causal link, should be considered.

Second, although the sample was drawn based on age and gender group allocations, it was limited to individuals willing to participate in a web-based survey, which may have introduced sampling bias. It is likely that individuals with severely poor health, less motivation to participate in web-based surveys, or heavy workloads and limited free time were underrepresented.

Third, this analysis excluded individuals who were employed before the pandemic but unemployed at the time of the survey, due to the small number of such individuals (n=17), which made meaningful analysis difficult. Therefore, people who lost their jobs due to the pandemic were not considered. Possible reasons for this exclusion include a lack of time or resources among the unemployed to participate or limited access to the internet. This study focused on individuals employed both before the pandemic and at the time of the survey, allowing it to demonstrate the severity of the mental health challenges faced by workers experiencing economic hardship.

### Conclusion

No previous studies have specifically addressed the factors that mitigate or amplify the negative impact of COVID-19-induced economic deterioration on mental health or explored gender differences in these effects. This paper analyzed data from a Japanese survey conducted during the second wave of the pandemic, a time when women’s mental health was believed to be in decline. The results revealed that the impact of economic deterioration was more pronounced when social support was limited, regardless of gender, and that negative interactions consistently showed significant main effects. These results suggest the ongoing need for targeted support for individuals with limited social support during societal crises such as a pandemic, irrespective of gender.

## Abbreviations

KUAS: Kyoto University of Advanced Science
NIBIOHN: National Institutes of Biomedical Innovation, Health, and Nutrition
OR: odds ratio
